# Health technology assessment capacity at national level in sub-Saharan Africa: an initial survey of stakeholders

**DOI:** 10.12688/f1000research.23263.1

**Published:** 2020-05-14

**Authors:** Samantha A. Hollingworth, Francis Ruiz, Mohamed Gad, Kalipso Chalkidou

**Affiliations:** 1 School of Pharmacy, University of Queensland, Brisbane, QLD, 4102, Australia; 2 School of Public Health, Imperial College London, London, UK

**Keywords:** Health technology assessment, Sub-Saharan Africa, survey, capacity building

## Abstract

**Background:** Health technology assessment (HTA) is an effective tool to support priority setting (PS) in health. Stakeholder groups need to understand HTA appropriate to their role and to interpret and critique the evidence produced. We aimed to rapidly assess current health system priorities and policy areas of demand for HTA in Sub-Saharan Africa, and identify key gaps in data and skills to inform targeted capacity building.

**Methods:** We revised an existing survey, delivered it to 357 participants, then analysed responses and explored key themes.

**Results:** There were 51 respondents (14%) across 14 countries. HTA was considered an important and valuable PS tool with a key role in the design of health benefits packages, clinical guideline development, and service improvement. Medicines were identified as a technology type that would especially benefit from the application of HTA. Using HTA to address safety issues (e.g. low-quality medicines) and value for money concerns was particularly highlighted. The perceived availability and accessibility of suitable local data to support HTA varied widely but was mostly considered inadequate and limited. Respondents also noted a need for training support in research methodology and data gathering.

**Conclusions:** While important in raising awareness of HTA as a tool for PS, this study had a low response rate, and that respondents were self-selected. A more refined survey will be developed to support engagement strategies and capacity building.

## Introduction

Many countries have committed to universal health coverage (UHC) in the context of the sustainable development goals ^1^ for affordable access to essential medicines and other health technologies. Many countries in sub-Saharan Africa (SSA) have established national health insurance systems (or are planning to) ^2^ but they require governments to set health priorities within fiscal limits ^3^.

Health technology assessment (HTA) provides a structured approach to synthesising evidence of clinical and cost effectiveness to inform priority-setting activities. Institutionalised HTA systems involve active participation from a range of stakeholders ^4^ including (government) decision makers, clinicians, academics, consumers, development partners, and HTA knowledge brokers ^5^. The International Decision Support Initiative (iDSI) is a global network of health, policy and economic expertise, which seeks to support countries make better decisions about efficient spending on healthcare. iDSI has been working in SSA since 2013 to develop local capacity and support implementation of robust HTA processes ^6, 7^. There is growing interest across the continent but the current HTA landscape is fragmented and undocumented. We aimed to assess the current health system priorities and policy areas that need HTA, the demand for HTA, and the supply of HTA efforts to identify gaps in data and skills.

## Methods

We used an existing framework to examine the need, demand, and supply of HTA in an anonymous survey comprising 12 questions ^8^. We used an online survey tool with purposive sampling; participants were contacted by email from the membership lists of the iDSI network and the African Health Economics and Policy Association (AfHEA). The survey was opened in June 2018. There were no inclusion or exclusion criteria regarding the participants. We analysed scale and ranking questions as the mean, and thematically analysed responses to open questions using an inductive approach ^9^.

### Consent

This survey was conducted in accordance with the Helsinki Declaration of 1975, as revised in 2008. All participants acknowledged written consent by starting the survey (as noted in the survey information section). No survey respondents can be identified in this paper as data has been aggregated. We do not have access to respondent’s details – data is fully anonymized. Respondents could leave the survey at any time. We used a scientific society distribution list to send emails to participants but we did not have access to the list.

## Results

Of the 357 recipients, 14% responded with 30 fully completing the survey. Half were from a research institute or university, or from within the Ministry of Health (27%). Respondents were from 14 countries but many were from Ghana (40%). The individual-level results are available as *Underlying data* ^9^.

### I. Need for HTA

In relation to the importance of particular attributes of HTA, respondents highlighted (mean rating out of 10): allocative efficiency (8.9), improving the quality of health care (8.8), transparency in decision making (8.4), budget control (8.0), and equity (7.9). They ranked the six main policy areas for which HTA was considered as urgently needed: 1) informing the design of the basic health benefits package (HBP); 2) producing clinical guidelines or disease management pathways; 3) informing the design of health service delivery; 4) coverage or reimbursement of individual health technologies; 5) provider payment or pay for performance schemes; and 6) registration of health technologies. A key consideration in HBP design was identifying services and technologies that should be covered but in a way that is financially sustainable.

Medicines were considered the most important technology type that would benefit from HTA approaches, followed by vaccines, public health programs, medical devices and diagnostics, service delivery initiatives or incentives, screening or referral programs, and other (e.g. surgical) interventions. Medicines were prioritised because of their relatively large budget impact and their use for many high-burden diseases. Improving the availability and management of vaccines would help to reduce the burden from communicable diseases.

Respondents emphasised three key decision problem areas that could be informed by HTA: health system financing; burden of disease considerations; and health service provision. Challenges in financing included inefficient financing structures (funding schemes and management); lack of funds; sustainability of national insurance; and the costs of medicines. In terms of burden of disease, respondents concerns included antibiotic resistance, and the impact of a growing dual burden of disease (communicable and non-communicable).

The respondents identified a need for research in: 1) health system financing (financing schemes, medicine pricing, and the design of sustainable essential benefits packages); 2) health service provision taking into account equity, efficiency, quality; 3) burden of disease (antibiotic medicine resistance, non-communicable disease, childhood immunisation); and 4) health policy research.

### II. Demand for HTA

The respondents provided information on who they considered to be the key users of HTA information and the types of HTA evidence most useful for decision making. The users were identified as: ministries of health; other government departments; public health insurance bodies; providers and health professionals; universities and research institutes; donor organisations; and pharmaceutical companies. The evidence considered most useful was: safety; economic issues; information on technology effectiveness; and accounting for social/ethical concerns. The safety concerns were explicitly linked to the availability and use of generic medicines where quality could not be guaranteed. The economics issues related to the tension between growing claims on, but limited availability of, health resources.

### III. Supply of HTA

All respondents were associated with organisations that generated or facilitated health services research. Research institutes highlighted their ability to provide expertise and skills for HTA research but some respondents noted a lack of human capacity for HTA. Political support was regarded as essential but could be impeded by politicised decision-making, internal politics in the leadership of the HTA process, cultural barriers in data and information sharing, and lack of funding for HTA activities.

The availability and accessibility of local data varied; generally, medicine prices were available but the costs of health services were neither easily known nor available. There are often data on the burden of disease but its application may be limited due to incomplete or unreliable documentation ^10^. The organisations which either generate or supply evidence mostly included government agencies, university and affiliated research institutes and donors plus development partners.

Respondents were asked to consider issues related to the wider ‘HTA infrastructure’ including the availability of existing process and methods manuals, or processes for involving the public in decision making. Respondents focused on the role of the public and civil society, and three themes emerged: 1) the extent of public involvement in consultation processes; 2) the role of advocacy; and 3) the absence of any public role in priority-setting decisions. Many respondents stressed the importance of consulting the public, but noted that in practice there was no involvement. Some respondents posited that such groups adopt an advocacy role by holding decision makers accountable, and creating pressure through media campaigns and other means to highlight dysfunctions in the health system.

Four main areas of training needs for HTA generators and users were identified: 1) research methods in HTA and data gathering (and economic evaluations); 2) identifying and implementing evidence and using it to inform policy; 3) conducting economic evaluations; and 4) developing capacity and building awareness. Some respondents noted that both HTA generators and health policy-makers and practitioners need training in HTA to facilitate the reliable and efficient interpretation and use of research results, translation into policy, and advocacy.

## Discussion

HTA was considered an important and valuable priority-setting tool with a key role in HBP design, clinical guideline development, and service improvement. Medicines and vaccines were the health technologies that would most benefit from HTA. The perceived availability and accessibility of suitable local data varied but was broadly considered inadequate and limited. There was a strong need for training support in research methodology and data gathering for HTA evidence.

This is, as far as the authors are aware, the first systematic survey of the HTA landscape in SSA. It is a preliminary survey; we will refine in future iterations. It appears that some questions were not entirely understood; this may have contributed to the low response rate. Despite this, we elicited in-depth responses from many respondents. They responded as individuals and so their views may not reflect those of particular agencies or governments.

Our results largely align with the WHO Global Survey on Health Technology Assessment by National Authorities ^11^ with regards to capacity needs, especially in the African region. The main barriers were a lack of qualified human resources, funding, and information ^11^. The interest in HTA as a priority-setting tool in the context of UHC is evident in the Asian region where HTA is more established than in SSA but there are ongoing challenges in the inconsistent use of HTA as means of updating benefits packages, concerns over transparency, and barriers related to data sharing ^12^.

The interest in using HTA to support priority-setting decisions for medicines and vaccines is welcome as these represent a high proportion of healthcare expenditure. Access to such technologies, particularly in the poorest countries, has been facilitated by international development partners but as the national income of aid-recipient countries increases, so do co-financing obligations. At some point countries will ‘graduate’ from aid – they will need mechanisms to effectively manage healthcare resources. Ghana has begun its journey to reduce reliance on donor assistance for health ^7, 13^.

Although HTA remains relatively under-developed in SSA, there is growing political commitment and policy interest ^14, 15^. Priority setting is inevitable: the question is not whether, but rather how, to set them. The HTA challenges outlined here could be mitigated by building HTA systems through pooling resources across countries and harmonising policies in health (e.g. medicines regulatory harmonisation, upstream of HTA ^16, 17^). The benefits of such harmonisation can be further enhanced through coordinated action on HTA policies which will help secure innovation uptake subsequent to regulation, at value-based prices reflecting local conditions.

## Data availability

### Underlying data

UQ eSpace: iDSI HTA survey SSA Data sharing. https://doi.org/10.14264/uql.2020.178 ^9^.

File ‘iDSI_HTA_survey_SSA_Data_sharing.csv’ contains individual-level responses from each survey respondent.

Data are available under the terms of the UQ Terms & Conditions: Permitted Re-Use with Acknowledgement.
