# Supplementary material for: Health technology assessment capacity at national level in sub-Saharan Africa: an initial survey of stakeholders
Source: F1000Res. 2021 Nov 11;9:364. Originally published 2020 May 14. [Version 2] doi: 10.12688/f1000research.23263.2 (PMC7309411; doi:10.12688/f1000research.23263.2)
Supplement: Peer review history

# Peer review: Reviewer response for version 1

**DOI:** 10.5256/f1000research.25683.r63459

**Published:** 2020-06-19

**Authors:** Rebecca Addo

**Affiliations:** 1 Centre for Health Economics Research and Evaluation, University of Technology Sydney, Broadway, NSW, Australia

This is a great article that used an online survey to explore the perception and views of SSA respondents in the need for HTA, demand for HTA and supply of HTA. Survey response rate was low, however responses were from participants across 14 countries in SSA thus fairly representative of the HTA landscape in SSA. Almost half of the respondents were from Ghana, where progress has been made towards the use of HTA in the health system. Respondents considered HTA was most importantly needed for informing the design of basic health benefits package (for national health insurance schemes) and development of clinical guidelines and disease management pathways. Stakeholders perceived as potential users of HTA were those from the ministry of health, public health insurance bodies. Whilst some respondents were of the view that they could provide expertise and skills for HTA, the majority were of the view that there were limited local human capacity, data and other infrastructures to support HTA.

 Specific comments to improving the research paper are summarised below:

1. The title of the article is suggestive of assessing capacity for HTA in terms of data, human and other infrastructure for HTA. However, the results are indicative of assessing what respondents perceived as the potential use, potential users and production of and barriers to producing HTA.
2. The abstract mentions 51 respondents completing the survey but the results note that only 30 fully completed. The authors should note whether partially completed surveys were included in the analysis or otherwise and provide justification for the choice.
3. It will be informative to provide a list of the countries that responded to the survey and also a list of where they worked.
4. Given that the majority of respondents were from Ghana, perhaps authors should provide some reasons/speculations as to why this was the case. Could it be as a result of Ghanaian respondents being more aware of HTA due to the recent involvement with iDSi projects for HTA and progress made towards using HTA for formal decision making in the country?
5. The authors should comment on whether the responses are likely to be biased by the general knowledge and awareness of respondents about HTA. Members of AfHEA and the majority of African health economists and policy analysts are mainly involved in health financing and not economic evaluation and HTA.
6. It will be interesting to know the differences in responses between respondents from Ghana countries that may have some ongoing work towards the use of HTA for health decision making and those that do not. Another area to consider is the differences in responses between respondents according to their area of work.
7. There are some studies from SSA that have explored the perceptions of decision-makers towards the use economic evaluations and HTA for decision making that covered the themes presented in this paper that the authors could have cited to support their findings. One study in Ghana (Addo *et al.*, 2020 ^1^)  and the other Ethiopia (Zegeye *et al.*, 2017 ^2^).
8. The discussion mentions Ghana transitioning from reliance upon donor assistance for health. Perhaps the authors should add more context and how that may contribute to the current landscape of HTA in Ghana.
9. Lastly, the authors mention refining the survey tool in future iterations as it appears some questions were not entirely understood. Was this limitation considered in the data analysis? This point should be noted as a limitation of the study as it may have affected the responses and subsequent results presented in this paper. Also the authors should note what 'refinement' they intend to make to the survey tool - is it content wise, or rewording, etc.

Is the work clearly and accurately presented and does it cite the current literature?

Partly

If applicable, is the statistical analysis and its interpretation appropriate?

Not applicable

Are all the source data underlying the results available to ensure full reproducibility?

Yes

Is the study design appropriate and is the work technically sound?

Yes

Are the conclusions drawn adequately supported by the results?

Yes

Are sufficient details of methods and analysis provided to allow replication by others?

Yes

Reviewer Expertise:

Health technology assessment, economic evaluation and quality of life.

I confirm that I have read this submission and believe that I have an appropriate level of expertise to confirm that it is of an acceptable scientific standard.

## References

[1] : The knowledge and attitude of Ghanaian decision-makers and researchers towards health technology assessment. *Soc Sci Med* .2020;250: 10.1016/j.socscimed.2020.112889 112889 10.1016/j.socscimed.2020.112889 32146238

[2] : Economic Evaluation in Ethiopian Healthcare Sector Decision Making: Perception, Practice and Barriers. *Appl Health Econ Health Policy* .2017;15(1) : 10.1007/s40258-016-0280-z 33-43 10.1007/s40258-016-0280-z 27637919

# Response

**Published:** 2021-11-02

**Authors:** Samantha A. Hollingworth

**Affiliations:** The University of Queensland, Australia

1. The title of the article is suggestive of assessing capacity for HTA in terms of data, human and other infrastructure for HTA. However, the results are indicative of assessing what respondents perceived as the potential use, potential users and production of and barriers to producing HTA.

**R: No changes made to the title.**

 2. The abstract mentions 51 respondents completing the survey but the results note that only 30 fully completed. The authors should note whether partially completed surveys were included in the analysis or otherwise and provide justification for the choice.

**R: Confirmed that there were 51 responses but only 30 were complete responses that were analysed. Text edited accordingly.**

 3. It will be informative to provide a list of the countries that responded to the survey and also a list of where they worked.

**R: Available in the dataset.**

 4. Given that the majority of respondents were from Ghana, perhaps authors should provide some reasons/speculations as to why this was the case. Could it be as a result of Ghanaian respondents being more aware of HTA due to the recent involvement with iDSi projects for HTA and progress made towards using HTA for formal decision making in the country?

**R: Yes.**

 5. The authors should comment on whether the responses are likely to be biased by the general knowledge and awareness of respondents about HTA. Members of AfHEA and the majority of African health economists and policy analysts are mainly involved in health financing and not economic evaluation and HTA.

**R: Text edited to “Some respondents may have known more about HTA than others which may have biased the results.”**

 6. It will be interesting to know the differences in responses between respondents from Ghana countries that may have some ongoing work towards the use of HTA for health decision making and those that do not. Another area to consider is the differences in responses between respondents according to their area of work.

**R: We agree. Please see dataset for more information. This could be explored in a later survey with more respondents.**

 7. There are some studies from SSA that have explored the perceptions of decision-makers towards the use economic evaluations and HTA for decision making that covered the themes presented in this paper that the authors could have cited to support their findings. One study in Ghana (Addo et al., 20201)  and the other Ethiopia (Zegeye et al., 20172).

**R: We have cited the suggested studies and included a further two studies:**

 Addo R, Hall J, Haas M, Goodall S: The knowledge and attitude of Ghanaian decision-makers and researchers towards health technology assessment. Soc Sci Med. 2020; 250:

 Zegeye EA, Mbonigaba J, Kaye SB, Wilkinson T: Economic Evaluation in Ethiopian Healthcare Sector Decision Making: Perception, Practice and Barriers. Appl Health Econ Health Policy. 2017; 15 (1): 33-43

 Hollingworth, S., Fenny, A. P., Yu, S.-Y., Ruiz, F., & Chalkidou, K. (2021). Health technology assessment in sub-Saharan Africa: a descriptive analysis and narrative synthesis. *Cost Effectiveness and Resource Allocation*, *19*(1), 39. https://doi.org/10.1186/s12962-021-00293-5

 Uzochukwu, B. S. C., Okeke, C., O'Brien, N., Ruiz, F., Sombie, I., & Hollingworth, S. (2020). Health technology assessment and priority setting for universal health coverage: a qualitative study of stakeholders' capacity, needs, policy areas of demand and perspectives in Nigeria. *Global Health*, *16*(1), 58. https://doi.org/10.1186/s12992-020-00583-2

# Peer review: Reviewer response for version 1

**DOI:** 10.5256/f1000research.25683.r63457

**Published:** 2020-05-18

**Authors:** Evidence Nyamadzawo

**Affiliations:** 1 HTAi, Johannesburg, South Africa; 2 ScHARR, University of Sheffield, Sheffield, UK

This article reports the results of an initial survey of Health Technology Assessment (HTA) stakeholders in sub-Saharan Africa (SSA). The authors aimed to identify where and how HTA methodologies can improve health system priority-setting and universal health coverage efforts at national level.

 The authors are experts on HTA and have a wealth of experience in priority-setting, health systems policy, and global health.

 In this interesting and important article, they have painted the current HTA landscape in SSA and highlighted areas in need of further investigations. Capacity-building efforts for HTA in SSA can now be refined based on the critical areas highlighted in this initial survey.

 The limitations of this survey have been acknowledged. I think the low response rate and incomplete answers are a reflection of the stage of development of HTA in SSA.

 Specific comments are provided below:

- The authorship of the article could have included African researchers currently on the continent on a full-time basis to help with local capacity development and international collaboration.
- Abstract: just semantics and preferences. "...tool to support.." may sound better as "...tool for supporting.."
- Methods: the authors state that "We used an existing framework.." I think they should have provided more information on this "existing survey". Was the survey used before and what were the findings?
- Ghana is mentioned as having given most of the responses and I know they have done a lot of work on local HTA use. The article could have given more detail on the developments in Ghana. It may help others to learn from such examples on the continent.

Is the work clearly and accurately presented and does it cite the current literature?

Yes

If applicable, is the statistical analysis and its interpretation appropriate?

Not applicable

Are all the source data underlying the results available to ensure full reproducibility?

Yes

Is the study design appropriate and is the work technically sound?

Yes

Are the conclusions drawn adequately supported by the results?

Yes

Are sufficient details of methods and analysis provided to allow replication by others?

Yes

Reviewer Expertise:

Maxillofacial and Oral Surgery Resident & Health Technology Assessment Researcher.

I confirm that I have read this submission and believe that I have an appropriate level of expertise to confirm that it is of an acceptable scientific standard.

# Response

**Published:** 2021-11-02

**Authors:** Samantha A. Hollingworth

**Affiliations:** The University of Queensland, Australia

1. The authorship of the article could have included African researchers currently on the continent on a full-time basis to help with local capacity development and international collaboration.

**R: Text inserted: potential collaborators were likely respondents.**

 2. Abstract: just semantics and preferences. "...tool to support.." may sound better as "...tool for supporting.."

**R: Done.**

 3. Methods: the authors state that "We used an existing framework.." I think they should have provided more information on this "existing survey". Was the survey used before and what were the findings?

**R: Cited a reference (#8).**

 4. Ghana is mentioned as having given most of the responses and I know they have done a lot of work on local HTA use. The article could have given more detail on the developments in Ghana. It may help others to learn from such examples on the continent.

**R: References to other countries inserted.**
